# Supplementary material for: Decline by design: Assessing decline policies as a decarbonisation strategy under the Paris Agreement
Source: PLoS One. 2025 Oct 24;20(10):e0334512. doi: 10.1371/journal.pone.0334512 (PMC12551923; doi:10.1371/journal.pone.0334512)
Supplement: S3 File — (PDF) [file pone.0334512.s003.pdf]

Supplementary information for

Decline by design: Assessing decline policies as a decarbonisation strategy under the Paris Agreement

Gregory Trencher[1], Mert Duygan[2,3] Adrian Rinscheid[4,] Daniel Rosenbloom[5]*, Peter Newell[6]

1. Kyoto University, Graduate School of Global Environmental Studies, Kyoto Japan
2. Centre for Development and Environment (CDE). University of Bern, Switzerland
3. Department of Environmental Social Sciences, Eawag, Dübendorf, Switzerland
4. Department of Political Science, University of St.Gallen, Switzerland
5. School of Public Policy and Administration, Carleton University, Canada
6. Department of International Relations, Sussex University, United Kingdom

* Corresponding author: daniel.rosenbloom@carleton.ca

Table S1. Examples of relevant and irrelevant decline policies

**Policy name and country**	**Text from Biennial Report or external source***	**Justification**
**Relevant decline policies (included)**		
Rail Electrification United Kingdom	'Major programme of rail electrification underway to replace older diesel trains with modern, low-emission electric trains. This means that operators are contractually obliged to meet emissions levels based on running modern electric rather than diesel traction.'	The policy describes a clear substitution effect, where old diesel trains are replaced with electric trains.
HFC-Phase-Down according to EU F-Gas-Regulation (PaM I.11) Germany	'The EU F-Gas Regulation 517/2014 features a phase-down scheme for the placing on the market (POM) of HFCs.'	The policy clearly describes a regulatory backed phase-down of a climate warming substance.
*Land use, land-use change and forestry sector emissions reduction pledge** Australia	'Measures include: ending native forest timber harvesting by 2030,'	The policy clearly describes a ban on forest harvesting, indicating that this practice will cease in 2030.
**Irrelevant decline policies (excluded ambiguous cases)**		
Fondo Rotativo Kyoto – "The Kyoto Fund" Italy	'The Kyoto Fund established by Law No. 296 of December 27, 2006, finances, through the granting of low interest loans, measures for the reduction of GHGs emissions. Article 9 of Decree Law No. 91 of June 24, 2014, allocated 350 million euros from the Fund to the energy upgrading of publicly owned school buildings, including kindergartens and universities. The energy efficiency interventions financed ensure an improvement of at least two "energy classes," corresponding to consumption savings of about 20-25 percent.'	The policy aims to improve energy efficiency in public buildings, leading to a reduction in energy consumption. However, the goal of increasing energy efficiency does not entail a commitment to abolishing the use of fossil fuels and associated technologies per se. Neither does this policy encompass an explicit substitution aim from which an inversely proportional decline effect could be anticipated.
*Capacity Mechanism** United Kingdom	'Increase in renewable energy (Energy supply), Switch to less carbon-intensive fuels (Energy supply)' '…the Capacity Market will ensure security of electricity supply by providing a payment for reliable sources of capacity, alongside their electricity revenues, to ensure they deliver energy when needed. This will encourage the investment we need to replace older power stations and provide backup for more intermittent and inflexible low carbon generation sources.' [Website]	The policy aims to improve the financial situation of frequently idled, back-up sources of electricity. Although the website text mentions an aspiration to 'replace older power stations', because new investments in back-up capacity are not limited to renewable energy, a clear decline of carbon-intensive assets or substitution of fossil fuels cannot be established.

*Sustainable Materials Management and Circular Economy** United States	'Provides a systemic approach to reduce the use of materials and their associated environmental impacts over their entire lifecycle.'	The measure involves a report from an expert group that lays out principles for shifting manufacturing towards circularity and sustainable materials. While a shift to circularity might reduce waste production, the direct effect that the presence of these design principles would have on waste production trends is highly uncertain and assumed to be weak.

Note: External sources consist exclusively of official policy documents or websites. If used, an external source is indicated in square brackets in the second column.

Table S2. Examples of justification behind decisions to classify policies as direct or indirect decline measures

Policy name and country	Text from Biennial Report or external source*	Justification
**Indirect decline policies**		
Increasing the share of RES in power generation Kazakhstan	'The goal is set in the Concept for the transition to green economy and in the Concept for FEC Development (updated at the moment). The target for 2030 was increased from 10% to 15% by the President of the Republic of Kazakhstan…'.	The target focuses on increasing the share of renewables, demonstrating a focus on the clean alternative. Decline of the incumbent technology (fossil fuels) would occur indirectly, as a result of substitution.
*Manitoba biofuel mandates** Canada	'As of January 1, 2022, Manitoba increased the minimum mandatory ethanol content in gasoline to 10% and the minimum mandatory biodiesel content in diesel fuel to 5%.'	The regulation focuses on increasing the share of renewables, demonstrating a focus on the clean alternative. Decline of the incumbent technology (fossil fuels) would occur indirectly, as a result of substitution.
**Direct decline policies**		
*Zero Emissions Transport Strategy 2022–30** Australia	'The strategy sets out policies that commit the ACT to phasing out light internal combustion engine vehicles from 2035, expanding the public EV charging network to ensure that there are at least 180 publicly available charging stations in the ACT'<br><br>'Prohibit onboarding of new internal combustion engine vehicles to rideshare and taxi networks by 2030' [ACT's Zero Emissions Vehicles Strategy 2022 – 30]	The policy describes a clear ambition to phase-out ICE vehicles in addition to goals of increasing ZEV sales. This demonstrates that the policy strategy includes aims and measures to directly target the incumbent technology.
49: Reduction of food losses (Act on the Prevention of Food Waste) Poland	'…mobilising the sellers of food to avoid its waste. It imposed on larger shops (with an area of more than 250 m2) the obligation to sign agreements with non-governmental organisations and the obligation to transfer unsold food, which is still fit for consumption, to these organisations free of charge. It also provides for penalties for wasting food which could have been used and for failure to sign the agreements.'	The policy clearly describes a principal ambition to directly reduce food waste, the incumbent practice.

Note: External sources consist exclusively of official policy documents or websites. If used, an external source is indicated in square brackets in the second column.

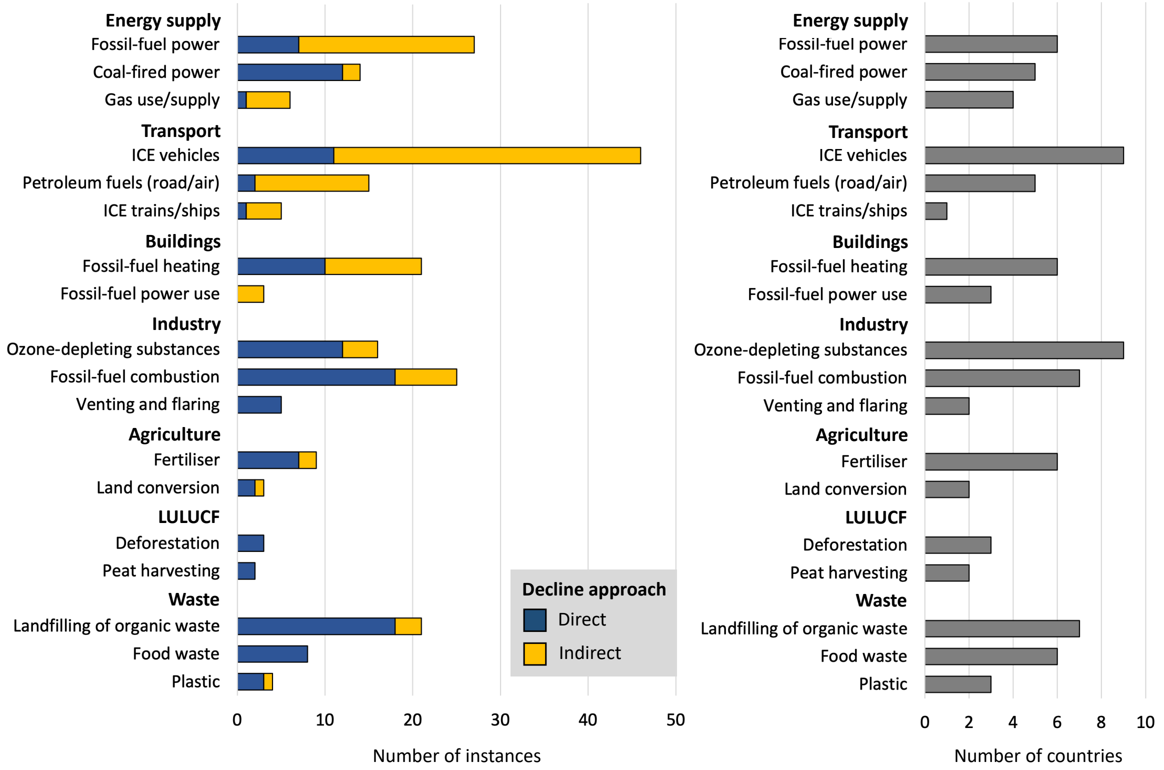

Figure S1. The most frequent targets of decline policy

Bars show the frequency of occurrence for a subset of the most common targets (n=233) out of a total of 251 in the dataset.

Table S3. Results of regression analysis in three statistical models

	(1) Partial model A		(2) Partial model B		(3) Full model	
Decline strategy (1 = direct)	0.798*	(0.398)			0.232	(0.316)
Decline intensity indicator (1 = high intensity)			3.374**	(0.992)	3.183**	(1.037)
Energy and buildings sector	-0.662	(0.483)	-1.160**	(0.334)	-1.112**	(0.359)
Transport sector	-1.632**	(0.512)	-1.762**	(0.465)	-1.696**	(0.421)
Industry sector	0.995	(1.078)	0.702	(1.095)	0.711	(1.079)
Waste sector	0.731	(0.712)	0.610	(0.924)	0.574	(0.921)
Constant	-4.288**	(0.904)	-5.856**	(1.285)	-5.880**	(1.226)
Log SD (Random Effect)	-0.190	(0.377)	-0.474	(0.432)	-0.443	(0.424)
Log SD (Residual Error)	0.631**	(0.093)	0.589**	(0.081)	0.586**	(0.081)
N		95		95		95

Shows regression coefficients from mixed-effects models with random effects modelled at the country level. Robust standard errors in parentheses. Dependent variable is the log-transformed share of avoided sectoral CO2 emission reduction estimates. Based on 95 decline policies for which estimates of mitigation impact were obtainable from Biennial Reports. A further three policies (not shown) were excluded due to implausibly high estimates that exceeded total annual sectoral emissions. The 'energy' category includes policies from the 'buildings' sector. Mitigation impact estimates for policies in the building sector were normalised in accord with the annual emissions of the energy sector because the former's emissions are not reported separately by countries. Statistical significance indicated as $^{+} p < 0.10$, $^{*} p < 0.05$, $^{**} p < 0.01$.

Table S4. Correlations of variables included in the regression analyses

	Decline strategy	Decline intensity	Energy sector	Transport sector	Industry sector
Decline strategy					
Decline intensity	.41				
Energy and buildings sector	-.04	.21			
Transport sector	-.26	-.19	-.32		
Industry sector	.07	.03	-.30	-.23	
Waste sector	.16	.05	-.26	-.20	-.19

The 'energy' category includes policies from the 'buildings' sector. Mitigation impact estimates for policies in the building sector were normalised in accord with the annual emissions of the energy sector because the former's emissions are not reported separately by countries.
