# Supplementary material for: Decline by design: Assessing decline policies as a decarbonisation strategy under the Paris Agreement
Source: PLoS One. 2025 Oct 24;20(10):e0334512. doi: 10.1371/journal.pone.0334512 (PMC12551923; doi:10.1371/journal.pone.0334512)
Supplement: Attachment — Submitted filename: Replies to reviewer comments.pdf [file pone.0334512.s006.pdf]

Review Comments to the Author

Reviewer #1

Thank you for the opportunity to review the manuscript "Decline by design: Assessing the role of policies that downscale carbon-intensive artefacts and activities under the Paris Agreement." The authors present a timely exploration of policies designed to reduce the production and use of carbon-intensive technologies across ten countries. They classify decline policies by type and analyze their expected impacts on emissions reductions. The manuscript is generally well written and tackles a highly relevant topic with clear policy significance. However, the manuscript has several important shortcomings that must be addressed before it can be considered for publication.

Reply➔ We thank you sincerely for the time and energy you have spent in reviewing our paper. We appreciate the detailed engagement with the manuscript and the methodological details. We have carried out the changes requested as below and are confident that the paper has been considerably improved as a result.

1. Unclear knowledge gap.

The authors claim to help bridge three knowledge gaps in the literature, but they simply list characteristics of previous studies that differ from theirs instead of a genuine knowledge gap. First, the authors mention that the literature on decline has mainly focused on historical cases. While I agree to some extent—some references (like [8], but also Plötz et al. 2018, and many others) address ongoing decline cases—it is unclear what can be learned that is new by looking at more recent cases. Second, what "advancement of knowledge" is hindered by the "lack of systematic and comprehensive analyses of large volumes of policies" (page 3)? What is the knowledge gap here? Third, I am not sure that because someone has not yet used a specific data source it can be considered a knowledge gap. The authors seem to simply repeat the same gap as they state in the previous paragraphs.
Put simply, what is the gap in our knowledge that your paper is bridging? What can be learned from using the new data and comparing policies across countries and sectors? I believe there is value in the research the authors present, but the knowledge gap has to be motivated much more strongly.

Plötz, P., Axsen, J., Funke, S.A. and Gnann, T., 2019. Designing car bans for sustainable transportation. Nature Sustainability, 2(7), pp.534-536.

Reply➔ We welcome the opportunity to clarify the nature of our contribution more precisely. We have reframed our summary of the gaps in the scientific literature around three themes:

- **Historical focus:** Most existing studies draw on historical cases where climate action was not central, leaving a gap in understanding contemporary, climate-driven decline strategies.
- **Limited scope:** Research remains narrowly focused on particular countries and sectors—especially energy—hindering generalisable insights into decline policies across diverse contexts and instruments.
- **Need for integrated views of decline and innovation:** There is a lack of empirical research on real-world policy mixes to explore how policymakers simultaneously use decline and innovation policies within integrated policy mixes to drive socio-technical transitions.
- **Policy effectiveness:** Limited attention has been given to how the specific design features of decline policies affect their actual mitigation effectiveness.

The full text version, modified in the introduction, is as follows:

Yet, while substantial progress has been made in elucidating the conditions, policies and processes that contribute to the decline of incumbent industries and technologies, academic work has stopped short of developing comprehensive and global analyses of contemporary decline policies. Indeed, many empirical investigations of decline have drawn from historical cases where climate change concerns were not the central driver of socio-technical change [29-33]. Moreover, the prevailing focus within the literature tends to centre on specific industries, countries or regions [4]. Energy and road transport have received the lion's share of attention, despite the importance of other domains such as waste, buildings, agriculture and land-use, as well as cross-cutting interventions [34]. Expanding the temporal, sectoral, and geographical scope of empirical research is, therefore, critical to deepen understanding of decline.

Beyond questions of scope, additional work is needed to elucidate the distinct role that decline-oriented measures play within policy mixes. Prior work has laid out important conceptual and theoretical foundations for understanding the potentially synergistic and accelerating effects of policy mixes that simultaneously promote the breakdown of the old and the buildup of the new in the context of pursuing socio-technical transformation and decarbonisation [5, 11, 28, 35, 36]. Nevertheless, empirical studies of actual policy mixes that adopt this dual view of innovation and decline remain somewhat limited [11, 37].

What is more, the effectiveness of different types of decline policies in reducing GHG emissions – whether assessed ex-ante through modelling or ex-post through empirical verification – has yet to be integrated within such a policy mix perspective on decline and innovation. Although energy system modellers have simulated the mitigation outcomes of various decline promoting policies such as carbon pricing, bans and mandates [38-40], there is still limited understanding of how specific design features – such as policy stringency, scope, timing and reduction ambition – influence decarbonisation effectiveness.

2. Conceptual Clarity.
In my opinion, the paper currently lacks a consistent and well-defined conceptual framework. Several key terms and categories remain ambiguous, hindering the manuscript's clarity and coherence. In particular:
2.1 Definition of "decline."
The authors refer to the "downscaling" of carbon-intensive technologies, materials, and processes, but they do not define what exactly is meant by "downscaling." This term requires a clear definition. To the best of my ability, the cited literature does not provide a definition either (e.g., refs 2, 3, 4, 7). What exactly is downscaling? Downscaling can be confused with moving from large-scale, centralized production to small-scale, distributed production. I would recommend that the authors stick to an established definition of decline.

Reply➔ We appreciate very much this observation, which prompted us to reconsider our use of the term "downscaling". In place, we decided to use **"reduction"** (to signify any degree of decline) and **"discontinuation"** (to signify a complete abolishment). This choice of words is based on our careful checking of the empirical material (the Biennial Reports), which shows that "reduction" is by far the most commonly used decline-related term.

We base our use of the term "discontinuation" on works such as the following:
Stegmaier, P., Kuhlmann, S., Visser, V.R., 2014. The discontinuation of socio-technical systems as a governance problem. The Governance of Systems Change.

Koretsky, Z. (2023). Dynamics of technological decline as socio-material unravelling. Technologies in Decline: Socio-technical approaches to discontinuation and destabilisation. Z. Koretsky, P. Stegmaier, B. Turnheim and H. van Lente. London and New York, Routledge: 13-40.

2.2 What declines?
The authors claim to expand the conception of what can be targeted by decline policies, but do so in a very confusing way, suggesting that technologies, substances, practices, and institutions are what can decline (page 5). Lacking a clear conceptual framework, it is difficult to understand how these terms differ from each other and how they are interrelated. From a conceptual perspective, the reader is left uncertain about the scope and boundaries of the analysis. This unclarity has subsequent effects when the authors refer to "incumbent elements" and "incumbent arrangements" that are equally unclear. I strongly suggest that the authors consider adopting one of the frameworks commonly used in the decline literature, like socio-technical systems, production and consumption systems, technological innovation systems, or others. Besides helping clarify what declines and allowing the authors to define and locate the different aspects of the system that decline policies can target, the use of a system-level framework would provide authors with an unambiguous vocabulary, for example, to talk about "incumbent regimes." Last but not least, a clear conceptual framework would help the authors to discuss more intelligibly the processes through which the policies they study achieve their effects. In repeated occasions throughout the text, the authors refer to the different "levers" that decline policies can use. However, without a conceptual framework that explains how policies affect the different components of the systems they try to influence, it is unclear what these levers are, how different policy instruments can act on them, or why some design features should be expected to be more effective than others.

Reply➔ We thank you for these comments, which have encouraged us to clarify our conceptual and empirical focus. We have sharpened our explanations as follows (page 5):

Drawing on this literature, we adopt a broad conceptualisation of deliberate decline, focusing on policies designed to reduce or eliminate one or more core elements of a carbon-intensive system. Consistent with prior research [3, 66], our empirical focus includes policies targeting elements such as *technologies and infrastructures* (e.g. ICE vehicles, fossil-fuel boilers, industry equipment), *substances and fuels* (e.g. plastics, ozone-depleting substances), *industry processes* (e.g. landfilling, logging, gas flaring) *and practices* (e.g. flying). Building on earlier conceptualisations of intentional decline [2, 67], we define a decline policy as 'a governance intervention intended or expected to induce the partial reduction or complete discontinuation of the production or use of one or more incumbent elements within a carbon-intensive system.'

Please note how our empirical focus has now been made explicit, since we explain that we focus on socio-technical elements such as technologies and infrastructures, substances and fuels, industry processes, practices and institutions, anchoring this conceptualisation in the relevant literature. In particular, the focus on these decline targets is supported by the study below:
Trencher, G., et al. (2023). "The evolution of "phase-out" as a bridging concept for sustainability: from pollution to climate change." One Earth 6.

2.3 Indirect decline
The conceptualization of indirect decline appears to be limited to substitution (e.g., promoting clean alternatives), but there could be other paths that lead to the reduction of the production and use of a technology without a direct substitution. For example, policies promoting working from home can lead to a reduction in the use of fuel because people travel less without supporting a cleaner alternative. Similarly, policies aimed at energy conservation can lead to a reduction in the use of fuel. Policies for building renovations could reduce the use of fossil fuels by improving insulation, even if not necessarily supporting a cleaner heating fuel alternative. Some of these policies appear in your dataset (e.g., policy 92) but do not seem to fit in your current description of indirect decline (on page 7). Some policies might even fall somewhere in between, for instance, how do you deal with efficiency or emission standards? If an efficiency or emission standard is set so high that

the incumbent technology cannot meet it, does it classify as a direct or indirect decline policy?

Reply➔ We thank you for this important comment about our conceptualisation of “indirect decline”. Your concerns about the lack of clarity about the type of policies that we include in our analysis and the types we exclude has provided an important opportunity for us to sharpen our explanations about this point. In sum, we have taken out the following measures to address your concern as follows:

1. Improved **Figure** 1 showing indirect and indirect decline pathways to make it clear that indirect decline policies embody a “substitution” function, which excludes many types of “regular” diffusion policies
2. Added a table showing the empirical scope of our study.
3. Improved our textual explanations in the background Section 2.1
4. Added tables of relevant (included) and irrelevant (excluded) policies in the supplementary material.
5. Explained the limitations of our method, emphasizing that countries would certainly possess more decline policies than those included in their Biennial Reports submitted to the UNFCCC.

We now explain these improvements in detail below:

1. Improvement of Figure 1

The figure has been improved to appear as follows. This now reflects clearly our emphasis on “substitution” – i.e. replacing an incumbent element with a new one – as a requirement for consideration as an indirect policy. This allows us to conceptually exclude many types of diffusion policies or energy efficiency policies that do not explicitly pursue a substitutive function.

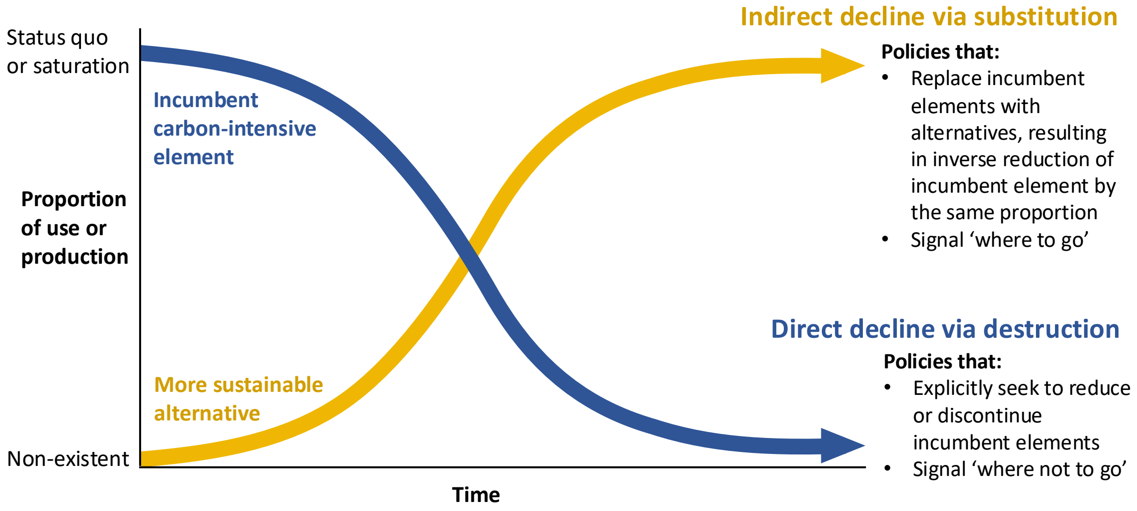

Figure 1. Direct decline and indirect decline policies

2. Table showing the empirical scope of our study

Your comment raises the important point that an extremely large number of policies could be expected to induce some degree of decline in a socio-technical system. Instead of arguing that there is a black line dividing relevant and irrelevant policies, we instead emphasise that the potential decline effect of some types of policies is more certain than other types. To achieve this, we added Table 1 below to our paper. Note how it uses the examples you raised in your comment and how it operationalises the logic conveyed in Figure 1.

Table 1. Empirical scope of study

Encompass an explicitly destructive or substitutive function?	Direct (decline through destruction)	Indirect (decline through substitution)
**Empirical focus (included)**		
Yes	• Bans, moratoria<br>• Phase-out programmes<br>• Restrictions on production/use<br>• Environmental standards<br>• Reduction goals (reducing landfill waste or fertilizer by 30%)<br>• Carbon pricing (taxes, emissions trading)	• Technology replacement program (diesel to electric trains/ships)<br>• Subsidies for fuel switching (gas to electricity) and equipment replacement (oil burners to heat pumps)<br>• Technology upscaling targets (50% renewables by 2030)<br>• Biofuel blending mandates (10% by 2025)
**Beyond empirical focus (excluded)**		
No	Not applicable	• Energy efficiency upgrades<br>• Fuel efficiency standards<br>• Subsidies to promote ZEVs or renewable energy installation without a replacement

	requirement<br>• General emissions reduction targets<br>• Investments in public transport to encourage modal shift<br>• Building energy efficiency labels

Regarding the specific examples you raise in your comment, our responses to these are as follows. Please note that this table below is **<u>not</u>** included in the manuscript:

Examples raised by reviewer (table not included in manuscript)

	**Reviewer's reasoning**	**Our logic and inclusion decision**
Policies promoting working from home	Can lead to a reduction in the use of fuel because people travel less without supporting a cleaner alternative.	Include as "direct decline" if there is a an explicit ambition or numerical target to reduce road traffic/car use.<br>Exclude if no ambition of target, and if description focuses on promoting home work.
Policies aimed at energy conservation	Can lead to a reduction in the use of fuel.	Exclude policies focused on raising energy efficiency as decline effect is uncertain. As illustrated by the 'rebound effect', the main problem is that gains in energy efficiency risk being offset by increases in overall use. Include if an absolute fuel reduction target is mentioned (e.g. reduce city fuel consumption by 20%).
Building renovations	Could reduce the use of fossil fuels by improving insulation	See reasons cited above. Exclude if no focus on replacing fossil fuels or fossil fuel equipment. Include as 'indirect' decline if policy includes explicit goal to replace fossil-fuel boilers.
Efficiency or emission standards	If an efficiency or emission standard is set so high that the incumbent technology cannot meet it, does it classify as a direct or indirect decline policy?	All carbon pricing schemes (carbon taxes, emissions trading) are included as 'direct' decline, as they take effect on the incumbent technology or fuel. This inclusion follows the conceptualisation of Kivimaa and Kern (2016), who cite carbon pricing as a 'destructive' policy. In general, emissions standards are not included if they are suggested in a way allowing the incumbent technology/fuel to continue. If there is explicit evidence of a downscaling ambition (e.g. the case of CO2 emission restrictions for ICEs in Europe) we include as 'direct decline'.

Please note that for the sake of preserving conceptual clarity, we do not include all policies that might contribute to a decrease in fossil fuel use or emissions as this

would open to the door to almost anything and defeat the purpose of our empirical focus on policies with a more certain decline effect.

3. Improve our textual explanations in the background Section 2.1
We have sharpened our textual explanations as follows. Please note that your observation that "indirect decline" occurs mainly via substitution is correct and aligns with our original intention (page 7).

But not every diffusion policy can be considered an indirect decline policy. This is because many leave room for the uninterrupted continuation of incumbent trajectories, especially in expanding markets where relative decarbonisation gains from a new technology's diffusion can be offset by the absolute growth of the incumbent system. One mechanism that can undermine decline outcomes is layering, when a new technology, substance or practice diffuses without displacing or substituting the incumbent element to a comparable degree [73, 74]. The case of consumer subsidies for ZEVs provides a compelling example. In some jurisdictions and contexts, ZEV-promoting policies have encouraged the purchase of a second vehicle, thus failing to reduce the size of the existing fleet of traditional ICE vehicles [75, 76]. A related mechanism is rebound, when per-unit efficiency gains are negated by increased overall use of the incumbent system element. This is particularly relevant for energy-efficiency policies, where rebound effects have been observed to erase gains in reducing fossil-fuel consumption in various domains. In the household sector, this occurs when occupants increase indoor temperatures on their thermostats or when larger dwellings are built [77, 78]. Similarly, in the transport sector, many hybrid vehicle owners respond to reduced mile-per-gallon costs by driving longer distances [79].

The aforementioned concerns about layering and rebound suggest that the potential of certain policy types to reduce the relative share of the incumbent element – whether directly or indirectly – is less certain than other policy types, ceteris paribus. Accordingly, as illustrated in Table 1, we limit our empirical scope in this study to policy types where a decline effect can be anticipated with a high degree of certainty. In doing so, we exclude policies such as energy efficiency upgrades and standards, purchase subsidies without replacement requirements, and broad emissions-reduction goals. By narrowing our empirical scope to policies that explicitly perform a destructive or substitutive function, our conception of two decline approaches distinguishes itself from other work [11, 28, 80] that take interest in all types of innovation and diffusion policies.

4. Add tables of relevant (included) and irrelevant (excluded) policies in the supplementary material.

We have added some examples of ambiguous indirect decline policies to our supplementary material to increase transparency around our coding logic with the following table:

Table S1. Examples of relevant and irrelevant decline policies

Policy name and country	Text from Biennial Report or external source*	Justification
**Relevant decline policies (included)**		
Rail Electrification United Kingdom	'Major programme of rail electrification underway to replace older diesel trains with modern, low-emission electric trains. This means that operators are contractually obliged to meet emissions levels based on running modern electric rather than diesel traction.'	The policy describes a clear substitution effect, where old diesel trains are replaced with electric trains.
HFC-Phase-Down according to EU F-Gas-Regulation (PaM I.11) Germany	'The EU F-Gas Regulation 517/2014 features a phase-down scheme for the placing on the market (POM) of HFCs.'	The policy clearly describes a regulatory backed phase-down of a climate warming substance.
Land use, land-use change and forestry sector emissions reduction pledge* Australia	'Measures include: ending native forest timber harvesting by 2030,'	The policy clearly describes a ban on forest harvesting, indicating that this practice will cease in 2030.
**Irrelevant decline policies (excluded ambiguous cases)**		
Fondo Rotativo Kyoto – "The Kyoto Fund" Italy	'The Kyoto Fund established by Law No. 296 of December 27, 2006, finances, through the granting of low interest loans, measures for the reduction of GHGs emissions. Article 9 of Decree Law No. 91 of June 24, 2014, allocated 350 million euros from the Fund to the energy upgrading of publicly owned school buildings, including kindergartens and universities. The energy efficiency interventions financed ensure an improvement of at least two "energy classes," corresponding to consumption savings of about 20-25 percent.'	The policy aims to improve energy efficiency in public buildings, leading to a reduction in energy consumption. However, the goal of increasing energy efficiency does not entail a commitment to abolishing the use of fossil fuels and associated technologies per se. Neither does this policy encompass an explicit substitution aim from which an inversely proportional decline effect could be anticipated.
Capacity Mechanism* United Kingdom	'Increase in renewable energy (Energy supply), Switch to less carbon-intensive fuels (Energy supply)'	The policy aims to improve the financial situation of frequently idled, back-up

	'…the Capacity Market will ensure security of electricity supply by providing a payment for reliable sources of capacity, alongside their electricity revenues, to ensure they deliver energy when needed. This will encourage the investment we need to replace older power stations and provide backup for more intermittent and inflexible low carbon generation sources.' [Website]	sources of electricity. Although the website text mentions an aspiration to 'replace older power stations', because new investments in back-up capacity are not limited to renewable energy, a clear decline of carbon-intensive assets or substitution of fossil fuels cannot be established.
Sustainable Materials Management and Circular Economy* United States	'Provides a systemic approach to reduce the use of materials and their associated environmental impacts over their entire lifecycle.'	The measure involves a report from an expert group that lays out principles for shifting manufacturing towards circularity and sustainable materials. While a shift to circularity might reduce waste production, the direct effect that the presence of these design principles would have on waste production trends is highly uncertain and assumed to be weak.

Note: External sources consist exclusively of official policy documents or websites. If used, an external source is indicated in square brackets in the second column.

Table S2. Examples of justification behind decisions behind direct and indirect decline policies

Policy name and country	Text from Biennial Report or external source*	Justification
**Indirect decline policies**		
Increasing the share of RES in power generation Kazakhstan	'The goal is set in the Concept for the transition to green economy and in the Concept for FEC Development (updated at the moment). The target for 2030 was increased from 10% to 15% by the President of the Republic of Kazakhstan…'.	The target focuses on increasing the share of renewables, demonstrating a focus on the clean alternative. Decline of the incumbent technology (fossil fuels) would occur indirectly, as a result of substitution.
Manitoba biofuel mandates* Canada	'As of January 1, 2022, Manitoba increased the minimum mandatory ethanol content in gasoline to 10% and the minimum mandatory biodiesel content in diesel fuel to 5%.'	The regulation focuses on increasing the share of renewables, demonstrating a focus on the clean alternative. Decline of the incumbent technology (fossil fuels) would occur indirectly, as a result of substitution.

Direct decline policies		
Zero Emissions Transport Strategy 2022–30* Australia	'The strategy sets out policies that commit the ACT to phasing out light internal combustion engine vehicles from 2035, expanding the public EV charging network to ensure that there are at least 180 publicly available charging stations in the ACT'<br><br>'Prohibit onboarding of new internal combustion engine vehicles to rideshare and taxi networks by 2030' [ACT's Zero Emissions Vehicles Strategy 2022 – 30]	The policy describes a clear ambition to phase-out ICE vehicles in addition to goals of increasing ZEV sales. This demonstrates that the policy strategy includes aims and measures to directly target the incumbent technology.
49: Reduction of food losses (Act on the Prevention of Food Waste) Poland	'…mobilising the sellers of food to avoid its waste. It imposed on larger shops (with an area of more than 250 m2) the obligation to sign agreements with non-governmental organisations and the obligation to transfer unsold food, which is still fit for consumption, to these organisations free of charge. It also provides for penalties for wasting food which could have been used and for failure to sign the agreements.'	The policy clearly describes a principal ambition to directly reduce food waste, the incumbent practice.

5. Limitations of our method

A final point is that our study relies on descriptions in Biennial Reports, which we supplement with publicly available information. We expect many other policies to also be relevant to decline. But we do not include as our study does not claim to exhaustively include all relevant policies in each country. We state this as a limitation with the following text on page 34.

In closing, two chief limitations of this study represent promising avenues for future research. First, the low number of countries and limited availability of estimates of mitigation impact in our sample point to the need for broader analyses covering more countries, sectors and policies. This study relied exclusively on policy descriptions contained in the Biennial Reports submitted to the UNFCCC as its chief data source. Since these reports are unlikely to comprehensively list the entire suite of a country's relevant decline policies – especially at the sub-national level – future work could extend our analytical procedure by drawing on additional data sources. These might include databases maintained by organisations such as IEA, IRENA and the NewClimate Institute as well as national or sub-national climate action plans.

3. Methodological concerns.

There are several aspects of the methodology that require clarification and more rigorous justification:

3.1 Potential selection bias?

On page 11, the authors state that policies using "decline-related terminology" were excluded if they were deemed to focus on diffusion or marginal decline. This approach appears arbitrary and could introduce selection bias. It might exclude policies that are introduced as decline measures but fail to achieve meaningful impacts. The authors should explain more clearly how policies were included or excluded from the sample. As a robustness check, it would be interesting to see a sample where any policies that explicitly mention a decline-related term in their description are added to the sample. With a list of the terms used to identify the policies.

Reply➔ Thank you for this comment. After clarifying our conceptual scope with the above responses, this statement no longer accurately reflects our verification procedure used during the policy screening stage. We thus changed this as follows (page 13):

ORIGINAL SUBMISSION

'Care was taken to exclude policies that, while described with decline-related terminology in the Biennial Report, were found upon closer inspection to ~~focus on diffusion or to include only marginal decline activities or objectives~~.'

REVISED SUBMISSION

'Care was taken to exclude policies that, while described with decline-related terminology (e.g. reduce fossil fuel use) in the Biennial Report, were found upon closer inspection to not correspond with our empirical focus, presented in Table 1.'

Please note that we do not adhere to an explicit list of decline terms (e.g. phase out, abolish, reduce, remove, prevent). We originally tried this in our earlier work, but we abandoned this 'manual text-mining approach' as we found that more accurate decisions about a policy's relevance could be made if reading all text in the Policies and Measures table and comparing with corresponding explanations in the Biennial Report and official external sources.

We do not preclude that our analysis has excluded some types of policies that

might exert a decline effect. We tackle this limitation by being more explicit about our empirical scope, explaining with transparency our focus on policies of which the decline effect is more certain (Table 1 in main text and above). We also do not deny the potential presence of a certain degree of selection bias. However, since we are now more clear about our empirical scope, we no longer see the need to include a robustness check that would indicate how many policies we excluded, especially the ambiguous ones.

3.2 Variable definitions.
The manuscript should clearly list: all sectors included in the study, the criteria used to classify policies as direct or indirect, all policy instruments considered, and their definitions. Each of these elements should be explicitly defined and justified. I could only find examples in the supplementary information rather than complete lists and definitions.

Reply➔ We thank you for this comment, which has prompted us to increase the transparency of our coding procedure and dataset. We have added Table 1 in the Methods to show the full range of variables used as follows:

Table 3. Coding variables used in database

Variable	Example or explanation	Source
Decline target (specific and aggregated)	• Specific (e.g. coal-fired power, heavy-duty ICEs, aviation fuel, gasoline, peat harvesting, deforestation)<br>• Aggregated (fossil-fuel power, fossil-fuel heating, gas use/supply, ozone-depleting substances, transportation fuels)	Inductively created.
Sector	• Energy supply<br>• Transport<br>• Buildings<br>• Industry<br>• Agriculture<br>• LULUCF<br>• Waste<br>• Cross-cutting	Categories defined by the UNFCCC, self-assigned by countries in their Biennial Report's *Policy and Measures* table.
Approach	• Direct decline<br>• Indirect decline	Inductively created, explained in Section 2.2.
Policy instrument	• Regulation<br>• Economic<br>• Fiscal<br>• Information and education<br>• Voluntary<br>• Research<br>• Other	Categories defined by UNFCCC, self-assigned by countries in their Biennial Report's *Policy and Measures* table.

Decline intensity	Score out of 0, 0.5 or 1 for seven policy design features:<br>• Strictness<br>• Numerical targets<br>• Reduction speed<br>• Geographic scale<br>• System coverage<br>• Reduction magnitude<br>• Stock turnover	Inductively created (**Table 4**).
Mitigation impact	105 kt CO_2-eq in 2030	Estimates of a policy's impact on annual emissions in a specific year, provided by countries in their Biennial Report's *Policy and Measures* table and supporting tabular data (Table 3).

Please note that three categories of codes (sector, policy instrument and mitigation impact) rely on codes or data provided by countries in their Biennial Report. That is, in the case of policy instruments, we do not code instruments ourselves. Instead, we simply take the classification provided by countries in their Biennial Report. Please note also that this table was originally included in the Supplementary Material but has been moved to the main paper to enhance transparency.

3.3 Data transparency.
The underlying data should be presented more transparently, ideally in tabular or supplementary format. This would improve reproducibility and allow readers to assess the robustness of the analysis more accurately. In particular, the authors must show correlation checks across the variables included in the regression analysis.

Reply➔ We share your concern for transparency and duly supply the datasheet of all coded policies, the codebook showing coding results, and the Stata code (**S1 and S2 files in Supplementary Data**) so that readers can reproduce the results obtained in this pilot study. For your information, Fig. 3, 4 and 6 were generated directly within the data file (Microsoft Excel). The regression analysis as well as Fig. 6 and 7 were produced using StataNow/MP 19.5. Please note also that the dataset was included in the original submission as an Excel file.

As requested, we have added the results of the correlation analysis as below in the supplementary data:

Table S4. Correlations of variables included in the regression analyses

	Decline approach	Decline intensity	Energy sector	Transport sector	Industry sector
Decline approach					
Decline intensity	.42				
Energy and buildings sector	-.04	.20			
Transport sector	-.26	-.20	-.32		
Industry sector	.07	.03	-.30	-.23	
Waste sector	.16	.05	-.26	-.30	-.19

The 'energy' category includes policies from the 'buildings' sector. Mitigation impact estimates for policies in the building sector were normalised in accord with the annual emissions of the energy sector because the former's emissions are not reported separately by countries.

We comment on the moderate correlation between decline approach and decline intensity in Section 4.3 of the manuscript.

As an additional measure, we have added mentions of policy names, countries and IDs in the following tables to increase transparency about what policies were used to formulate these findings:

Table 5a. Direct decline: Illustrative policies by sector

Sector	Decline target	Instrument examples	Policy examples (name and ID)*
Energy supply	One or more fossil fuel power sources.	• Targets, timelines and environmental standards to reduce or phase out coal power by a specific year.	• Germany's 'Act on the Phase-Out of Coal Plants' (127)<br>• Kazakhstan's 'Reducing the Share of Coal in Power Generation' (246)
Transport	ICE vehicles. Production or use of fossil fuels in road transport.	• Bans on the sale of new ICE vehicles.<br>• Vehicle registration tax based on CO2 emissions.<br>• ICE vehicle-free zones	• Germany's 'Stronger Weighting of the CO2 Component of the Vehicle Tax from 2021 Onwards' (140)<br>• United Kingdom's 'Regulation to Phase Out all New Non-Zero Emission Road Vehicles' (176)<br>• France's 'Low-Emission Zones (ZFE)' (216)
Buildings	On-site combustion of fossil-fuels in building equipment.	• Bans on installations of new fossil-fuel heating systems.<br>• Building codes setting rising limits on on-site fossil-fuel use.	• Germany's 'Building Energy Law (GEG)' (134)<br>• United State's 'Federal Building Rulemakings (203)<br>• Canada's (Québec) 'Chauffez Vert Program' (223)
Industry	Production/use of ozone-depleting substances.	• Mandatory targets to reduce ozone-depleting substance production.	• Australia's 'HFC Management – Regulations' (2)<br>• Poland's 'Proposed Adoption of

	Fossil-fuel combustion.	• Carbon pricing measures like carbon taxes or emissions trading.	More Stringent Requirements for the Limitation of the use of Fluorinated Greenhouse Gases' (241)<br>• Kazakhstan's 'Carbon Tax on Sectors not Covered by the Emissions Trading System' (248)
Agriculture	Fertiliser use.	• Voluntary or mandatory targets to reduce fertiliser use.	• Canada's 'Fertilizer (30% Reduction From 2020 Levels)' (107)<br>• Germany's 'Implementation of German Fertilisation Ordinance 2020' (144)
LULUCF	Deforestation or land use.	• Bans on deforestation<br>• Aspirational deadlines to end peat harvesting.	• Australia's (Victoria) 'Land use, Land-use Change and Forestry Sector Emissions Reduction Pledge' (28)<br>• Germany's 'Reduction of Peat as Substrate in Gardening' (147)<br>• United Kingdom's 'Forestry Act Felling Licence Regulations and Environmental Impact (Forestry) Regulations' (164)
Waste	Landfilling of organic or food waste.	• Ban landfilling of organic waste.<br>• Mandates to reduce food waste practices.	• Italy's 'Increase Separate Collection of Urban Waste' (232)<br>• Poland's 'Reduction of Food Losses (Act on the Prevention of Food Waste)' (243)

*ID numbers refer to specific policies listed in the codebook (see S1 Supplementary Data).

Table 5b. Indirect decline: Illustrative policies by sector

Sector	Decline target	Instrument examples	Policy examples (name and ID)*
Energy supply	One or more fossil fuel power sources.	• Aspirational or mandatory timelines or environmental standards to increase the share of renewable electricity.<br>• Renewable portfolio standards.	• Australia's (Northen Territory) 'Renewable energy target' (18)<br>• Japan's 'Renewables Obligation' (158)<br>• Kazakhstan's 'Increasing the Share of RES in Power Generation' (250).
Transport	ICE vehicles	• Aspirational or mandatory timelines or environmental standards to increase the share of ZEVs.<br>• Subsidies or vehicle replacement programmes in fleets.<br>• Mandatary targets for biofuel blending in petroleum fuels.	• Canada's 'Light-Duty Vehicles GHG and ZEV Sales Requirements' (74)<br>• Japan's 'Introduction of EV Waste Collection Vehicles' (114)<br>• United Kingdom's (Scotland) 'Vessel Replacement Programme' (189)<br>• Poland's 'Development of Electromobility (Bus Replacement)' (239)<br>• United Kingdom's 'Renewable Transport Fuel Obligation (RTFO)' (155)
Buildings	On-site combustion of fossil-fuels in building equipment.	• Subsidies for installations to replace fossil-fuel heating equipment.	• United Kingdom's 'Boiler Upgrade Scheme' (173)<br>• Poland's 'Warm Flat Priority Programme' (237)

Industry	Fossil-fuel combustion	• Subsidies for replacing existing equipment or fuel switching.	• Canada's 'Québec Bioenergy Program' (43)<br>• Japan's 'Promotion of Fuel Conversion' (113)<br>• Italy's 'Incentives to Biomethane and Other Advanced Biofuels' (229)
Agriculture	Fertiliser use or conventional agriculture.	• Subsidies or capacity building to support conversion to organic agriculture or to natural fertilisers.	• Germany's 'Increase of Organic Farming to 20% of Total Agricultural Utilised Land' (145)<br>• France's 'Agricultural Component of the Circular Economy Roadmap' (210)
LULUCF	None identified.	None identified	None identified
Waste	Reduce landfilling waste.	• Subsidies, targets or capacity building to divert landfilling by encouraging or mandating recycling.	• Australia's 'Food Waste for Healthy Soils Fund' (9)

*ID numbers refer to specific policies listed in the codebook (see S1 Supplementary Data).

3.4 Other methodological concerns.

• Comparing emission reductions in absolute terms across countries and sectors of widely ranging sizes can lead to misleading results. Should not relative changes in emissions should be used (e.g., (emissions in sector A of country B at year X – emissions in sector A of country B at year X+t with policy) / emissions in sector A of country B at year X)?

Reply➔ Thank you for this comment. We share your concern that comparing emissions reduction estimates across diverse sectors and countries with widely ranging economic sizes and energy systems risks creating misleading results. To address this concern, we have normalised each estimate of a policy's mitigation effect by dividing it by the total volume of emissions in each sector in 2019. Note that we only use one reference year (2019) when normalising emissions by sector, regardless of the year that a mitigation estimate refers to. This ensures a common baseline for all policies within our sample. Incidentally, as we explain in our methods, 2019 year was chosen as the baseline year for two reasons. First, it was the most recent year for which emissions data were available in the Fifth Biennial Reports at the time of publication (late 2022). Second, emissions in 2019 were unaffected by the global COVID-19 pandemic, which disrupted emissions patterns from 2020 onwards.

To illustrate our sectoral emissions normalisation procedure with an example, if

Policy A in Country α's energy sector was projected to reduce annual emissions by 150 kt-CO2 in 2035, we divide this by the total emissions for the energy sector in 2019 (e.g. 1500 kt-CO2). In this case, the policy would reduce the energy sector's emissions by 10% compared to the reference year (2019). To reflect this change, our figure showing the marginal means based on the regression analysis has been updated to show the average **share of avoided sectoral emissions** for each policy.

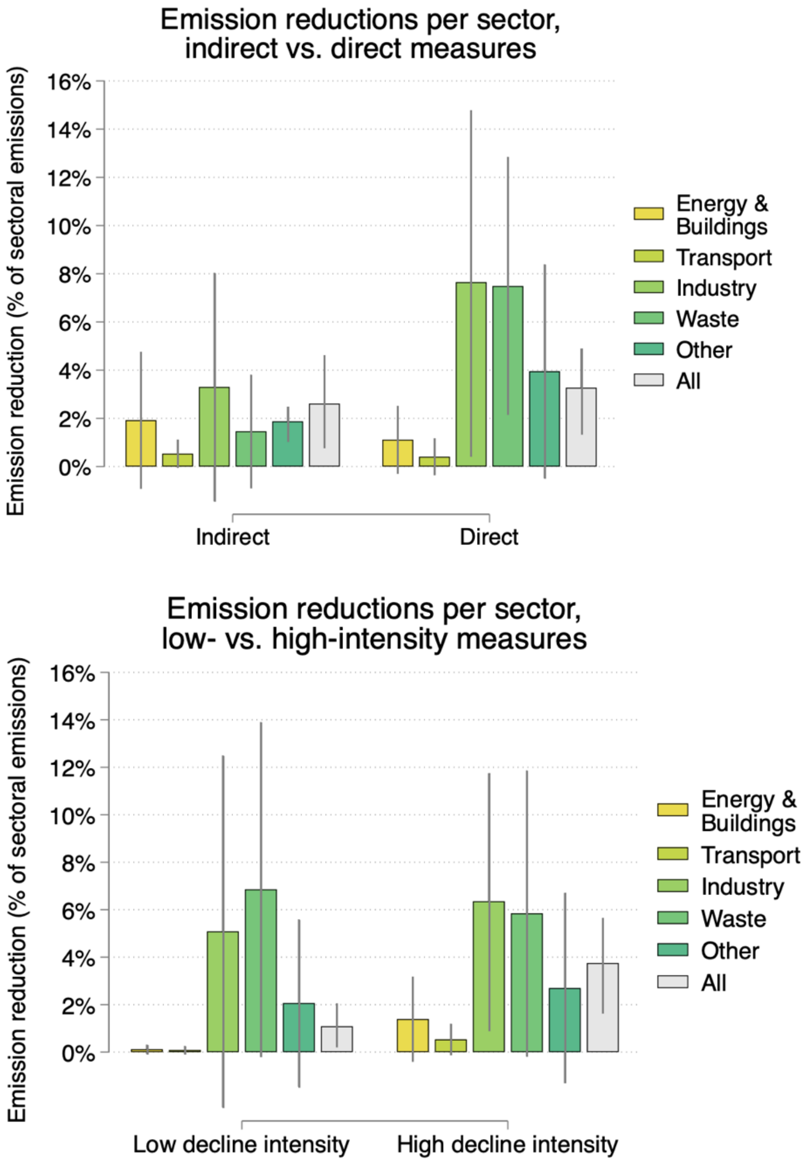

Fig 8. Marginal means of CO_2 reduction potential by sector and policy type

Bars show marginal means of the share of sectoral emissions avoided for 95 policies that included mitigation impact estimates in their Biennial Report, along with confidence intervals. Means are based on mixed-effects

models, with random effects at the country level and robust standard errors. To calculate the normalised emission reductions, we used the highest mitigation impact estimate provided for any year between 2020 and 2040 in each country's Biennial Report and divided this by that sector's total annual emissions in 2019. This method thereby reflects the peak mitigation potential of a policy irrespective of the year introduced. The 'energy' category includes policies from the 'buildings' sector. Mitigation impact estimates for policies in the building sector were normalised in accord with the annual emissions of the energy sector because the former's emissions are not reported separately by countries. The 'other' category includes sectors with too few estimates to derive marginal means (agriculture, LULUCF, cross-cutting). "All" represents the marginal mean obtained across all sectors. Decline intensity is categorised as 'low' (≤0.5) or 'high (>0.5) based on intensity scores, which reflect the unweighted average of seven indicators (**Table 4**).

Please note that we take the highest annual emissions reduction estimate for any year. For example, if Policy A was estimated to reduce annual emissions by 100 t-CO2 in 2025, 110 t-CO2 in 2030 and 150 t-CO2 in 2035, we would take only the latest value as the basis for our analysis. This decision is justified by our interest in the maximum mitigation potential of each policy regardless of the timing when this occurs. We added explanations about this process and logic to our Methods section as follows (page 16):

The dependent variable in the regression is the projected mitigation impact of each policy, normalised as a share of total annual emissions in the corresponding sector in 2019. This year was chosen for two reasons. First, it was the most recent year for which emissions data were available in the Fifth Biennial Reports at the time of publication (late 2022). Second, emissions in 2019 were unaffected by the global COVID-19 pandemic, which disrupted emissions patterns from 2020 onwards.

This normalisation was necessary to address substantial variation in the estimates of each policy's mitigation potential and to enable more meaningful comparisons of decarbonisation effectiveness across sectors compared to using unnormalised absolute values. Because the distribution of projected mitigation impacts was highly skewed due to a small number of policies with disproportionately large estimates (ranging between 20–65% of sectoral emissions), we log-transformed the normalised values of avoided sectoral emissions. Due to our interest in the peak mitigation potential of policies irrespective of the timing when this occurs, we used the highest value listed for the policies that provided estimates for multiple years. To account for the hierarchical structure of the data, we applied mixed-effects models with random effects specified at the country level. Finally, to illustrate emission reduction trends in individual sectors, we derived marginal means from the regression results.

Why are differences between countries never shown in figures or tables?

Reply➔ We appreciate this question. The main reason is that our n-size is small and thus would not allow a meaningful analysis of country trends, especially if trying to identify trends based on groups of countries and common socio-political conditions. However, in our ongoing research, we are currently coding all Annex 1 countries (n=39) and plan to identify trends across groups of countries. However, in this first paper, which is an exploratory study intended to pilot our approach and conceptualisation of decline, we have decided to emit a full-fledged analysis of country trends. This said, we decided to add one figure in the main text showing the proportion of direct decline and indirect decline policies by country, as follows:

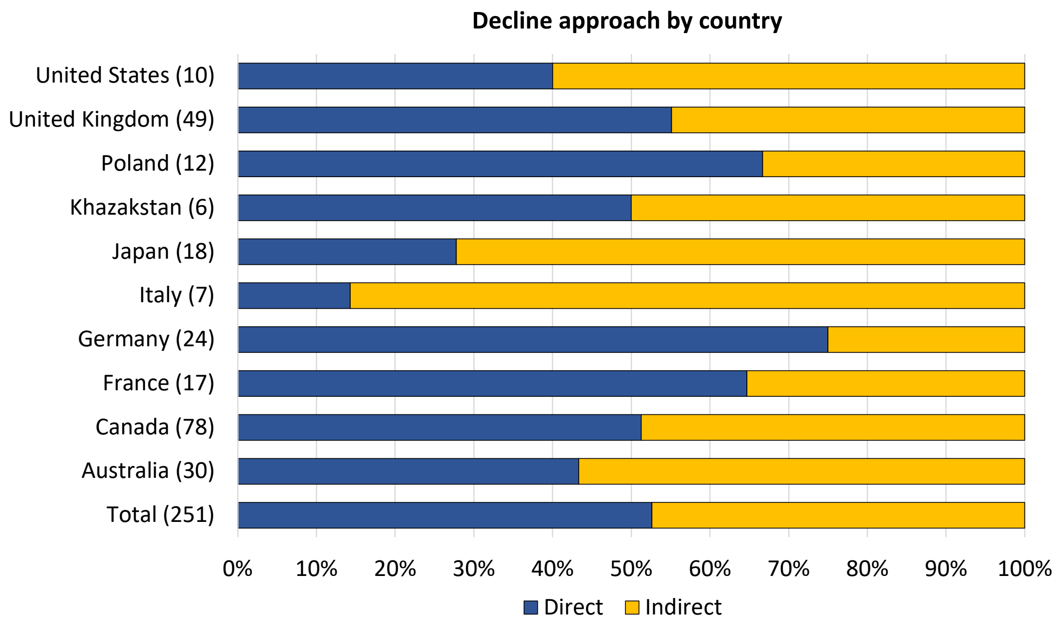

Fig 3. Distribution of policies across sectors and countries by decline approach

We explain this figure as follows in the main text:

Although our analysis did not explicitly aim to identify country-level trends due to the limited size of the Annex I group selected for analysis (n=10), our findings show that some countries predominantly employ direct decline policies whereas others lean more towards indirect approaches (Figure 3b). The three countries with the highest use of direct policies are Germany, Poland and France. Among these, Germany stands out, with more than 75% of its decline policies directly targeting incumbent elements, especially in transport, energy and industry. In contrast, Italy, Japan and the US exhibit a strong inclination towards

indirect approaches.

We also added this following text to the conclusion, in the discussion of limitations: Second, an important opportunity remains to investigate how political economy conditions shape a country's adoption and implementation of decline policies as a decarbonisation strategy. While our analysis revealed that some countries such as Germany feature a relatively high proportion of direct decline policies in their Biennial Reports, it did not explore the contextual reasons behind the cross-national variation in the shares of direct and indirect approaches. Similarly, questions remain about the conditions that affect the ability of countries to design and implement high intensity decline policies. Clarifying the political economy factors that shape the variations observed in policy characteristics would be an important way to enhance our understanding of the conditions needed to achieve politically accelerated decline.

Figure 6 provides very little information. Show the distribution of values with boxplots or a similar graph.

Reply➔ We agree that a bar chart of arithmetic means is not the best way to convey the information in our dataset. Yet a boxplot or similar graph is problematic for binary or categorical variables. Instead, we have now built a jitter plot which shows the distribution for all 8 indicators conditional on decline type, along with arithmetic means. We think that this new version of the figure is a considerable improvement.

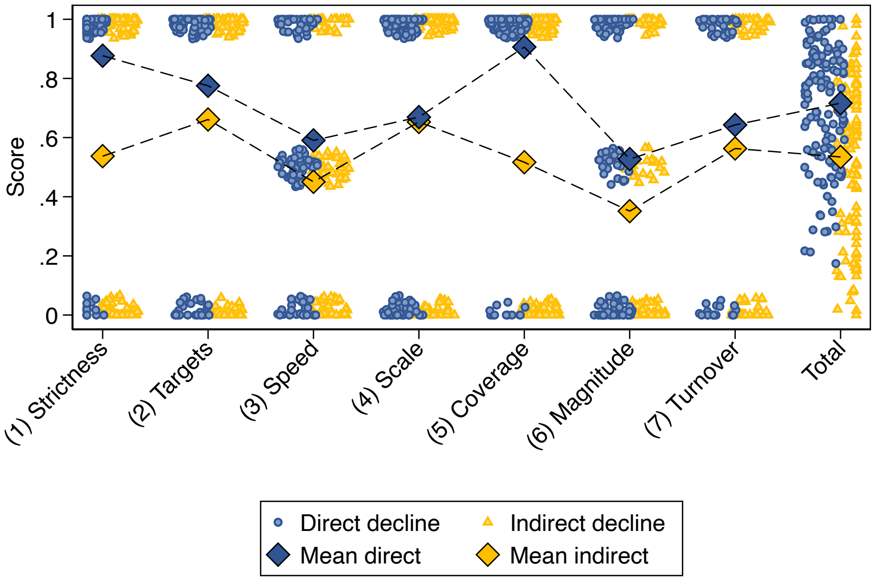

Fig 5. Decline intensity of direct and indirect approaches

Figure 7 seems to mix direct and indirect policies.

Reply➔ Your comment refers to the figure below. We do not make a separate version for direct and indirect decline policies as the n-size of our sample (235 policies and 251 measures) is not sufficient to provide representative claims about the intensity of decline approaches for each target. Consequently, if splitting this figure into direct and indirect decline, the n-size for many targets in LUCUF and Agriculture, for example, would shrink to 1, 2 or 3. We hope this clarifies why we have not broken this figure down into direct/indirect decline.

	(1) Strictness	(2) Numerical targets	(3) Reduction speed	(4) Geographic scale	(5) System coverage	(6) Reduction magnitude	(7) Stock turnover*	Total decline intensity	No. of policies
**Energy supply**									
Fossil-fuel power	0.70	0.74	0.59	0.44	0.81	0.41	0.74	0.64	27
Coal-fired power	1.00	1.00	0.86	0.43	1.00	0.79	1.00	0.87	14
Gas use/supply	0.83	0.50	0.25	0.67	0.83	0.17	0.33	0.56	6
**Transport**									
ICE vehicles	0.61	0.83	0.65	0.65	0.41	0.63	0.26	0.59	46
Petroleum fuels (road/air)	0.80	0.80	0.40	0.73	0.93	0.03	-	0.62	15
ICE trains/ships	0.40	0.80	0.40	1.00	0.80	0.70	0.60	0.67	5
**Buildings**									
Fossil-fuel heating	0.57	0.48	0.36	0.67	0.48	0.45	0.43	0.50	21
Fossil-fuel power use	0.33	1.00	1.00	1.00	0.00	1.00	0.33	0.70	3
**Industry**									
Ozone-depleting substances	0.88	0.69	0.38	1.00	0.81	0.38	-	0.69	16
Fossil-fuel combustion	0.84	0.52	0.30	0.52	0.88	0.10	0.72	0.57	25
Venting and flaring	1.00	0.60	0.40	0.20	0.80	0.20	-	0.60	5
**Agriculture**									
Fertiliser	0.44	0.33	0.17	1.00	0.78	0.00	-	0.45	9
Land conversion	0.33	0.67	0.50	0.67	0.33	0.33	-	0.47	3
**LULUCF**									
Deforestation	1.00	0.67	1.00	0.67	0.67	0.67	-	0.86	3
Peat harvesting	0.00	1.00	0.50	1.00	0.50	1.00	-	0.73	2
**Waste**									
Landfilling of organic waste	0.81	0.90	0.62	0.52	0.90	0.48	-	0.70	21
Food waste	0.50	0.75	0.56	0.75	0.88	0.50	-	0.66	8
Plastic	1.00	0.75	0.50	1.00	0.50	0.50	-	0.71	4

Fig 6. Evaluation of decline intensity for most common decline targets

Figure 8 must show the uncertainty interval around the estimates.

We have included the uncertainty intervals as requested. Please see Figure 8 pasted above. Please note that we had previously included the whiskered version showing uncertainty intervals in the supplementary material, but have since decided to display them in the main paper.

4. Policy types

While the policy types sound relatively convincing, an analysis of the expected emission reductions suggests that there is no statistically significant difference between them (except between types 1 and 4). This makes me think that the authors may be overstating the relevance of their distinction or that further thinking or analysis must go into finding categories to differentiate decline policies that align better with their expected effectiveness. Here, I used absolute CO2 emissions reductions, but I believe the authors should instead use relative emissions reductions by sector and country. (I copy the code from Python in case they want to reproduce the test, a figure and a table in the attached PDF)

Reply➔ We appreciate the time you have taken to prepare this analysis. We interpret your comment as a critique of our decision to include the table below and the associated text as an empirically informed typology.

Table 6. A typology of decline policies and expected effect

		**Decline intensity**	
		High	Low
**Decline approach**	Direct (decline through destruction)	**Type 1:** Abolishes the old, signalling where not to go, leveraging strictness, explicit targets, coverage and speed.<br><br>Example policies (ID): coal phase-out or emission regulations (66, 68, 127, 227); fossil-heating installation bans (93, 170); emissions trading schemes (130, 169); logging bans (28).	**Type 3:** Reduces the old, but with limited strictness, reduction ambitions, scale, speed or coverage.<br><br>Example policies (ID): carbon taxes (38, 220); fertiliser reduction support (121, 210); food waste reduction programmes (126).
	Indirect (decline through substitution)	**Type 2:** Substitutes old with new, signalling where to go, leveraging strictness, explicit targets, coverage and speed.<br><br>Example policies (ID): ZEV sale targets (75, 115); renewable energy penetration targets (3, 24, 37, 250).	**Type 4:** Substitutes old with new, but with limited strictness, reduction ambitions, scale, speed or coverage.<br><br>Example policies (ID): biomethane grid injection programmes (183); ICE vehicle replacement programmes (84, 114, 231); fuel switching subsidies (35, 43, 113).

ID numbers refer to specific policies listed in the codebook (see S1 Supplementary Data).

After debating the validity of this typology across our team, we have decided to retain this table and discussion in the paper for the reason that we do not attempt to demonstrate the relevance of this categorisation from the perspective of decarbonisation impact. The analysis of decarbonisation impact was carried out based on the regression analysis. In contrast, the goal of the section on policy types is to qualitatively describe, based on insights obtained from the literature, the defining characteristics and strengths/weaknesses of the four policy types. Our focus on the decline approach (direct/indirect) and decline intensity (low to high) is justified by the significance of these features in our dataset.

We have clarified this intent with the addition of the following text at the location where we present the typology (**page 29**):

We underscore the qualitative nature of this typology. Unlike the above regression analysis, we do not set numerical thresholds here to distinguish 'high' from 'low' intensity. Furthermore, this classification is not derived from the dependent variable (mitigation impact), but rather from the two dependent variables (decline approach and decline intensity). As the preceding analysis demonstrates, these last two dimensions are not only highly valuable for characterising the decline policies in our dataset, but they also hold critical relevance for decarbonisation impacts. This said, since the primary goal of this exercise lies in capturing the diversity of decline policies designed by governments, we do not evaluate the emissions reduction effectiveness of each archetype. We leave this task to future research, ideally supported by a larger sample of policies and emissions reduction estimates.
